# Abrupt termination of vitamin C from ICU patients may increase mortality: secondary analysis of the LOVIT trial

**DOI:** 10.1038/s41430-022-01254-8

**Published:** 2022-12-20

**Authors:** Harri Hemilä, Elizabeth Chalker

**Affiliations:** 1 Department of Public Health, University of Helsinki, POB 20, FI 00014 Helsinki, Finland; 2 Biological Data Science Institute, Australian National University, Canberra, ACT 2601 Australia

**Keywords:** Nutrition therapy, Drug development

## Abstract

**Background:**

The LOVIT trial examined the effect of vitamin C on sepsis patients, and concluded that in adults with sepsis receiving vasopressor therapy in the ICU, those who received 4-day intravenous vitamin C had a higher risk of death or persistent organ dysfunction at 28 days than those who received placebo. The aim of this study was to determine whether the abrupt termination of vitamin C administration could explain the increased mortality in the vitamin C group.

**Methods:**

We used Cox regression with two time periods to model the distribution of deaths over the first 11 days in the LOVIT trial.

**Results:**

Compared with a uniform difference between vitamin C and placebo groups over the 11-day follow-up period, addition of a separate vitamin C effect starting from day 5 improved the fit of the Cox model (*p* = 0.026). There was no difference in mortality between the groups during the 4-day vitamin C administration with RR = 0.97 (95% CI: 0.65–1.44). During the week after the sudden termination of vitamin C, there were 57 deaths in the vitamin C group, but only 32 deaths in the placebo group, with RR = 1.9 (95% CI: 1.2–2.9; *p* = 0.004).

**Conclusion:**

The increased mortality in the vitamin C group in the LOVIT trial is not explained by ongoing vitamin C administration, but by the abrupt termination of vitamin C. The LOVIT trial findings should not be interpreted as evidence against vitamin C therapy for critically ill patients.

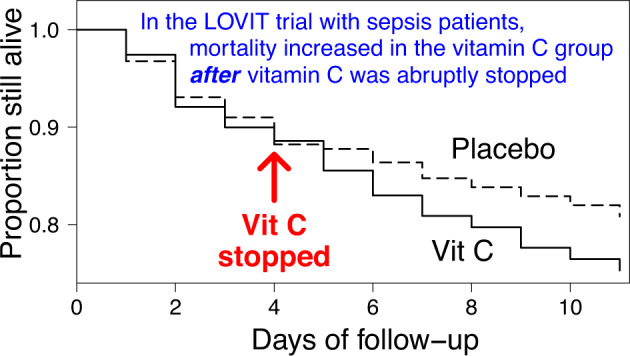

## Introduction

There is evidence from randomized trials that vitamin C may be beneficial for critically ill patients. Oral and intravenous vitamin C shortened the length of ICU stay on average by 7.8% in a meta-analysis of 12 trials with 1766 patients [1]. In another meta-analysis of 8 trials with 685 patients, vitamin C reduced the length of ventilation time, and meta-regression indicated that the effect was significantly greater in patients with more severe medical conditions [2].

The potential benefits of vitamin C for sepsis have been reviewed [3]. In animal studies of sepsis, vitamin C has prevented hypotension and lung injury, improved capillary blood flow, and prolonged survival [3–5]. The most common cause of sepsis is pneumonia [6, 7]. Vitamin C deficiency increases the risk of pneumonia and there are indications that vitamin C could be beneficial against pneumonia [8]. Sepsis is often associated with myocardial dysfunction [6, 7], and meta-analyses indicate that vitamin C may protect the heart when under stress [9, 10].

Two randomized trials found that vitamin C decreased mortality due to sepsis [11, 12]. In our reanalysis of the CITRIS-ALI trial with sepsis patients [12], we showed significant time-dependent modification of the vitamin C effect, with decreased mortality during the 4-days of vitamin administration [13]. The vitamin and placebo groups did not however differ thereafter, indicating that the length of time of vitamin C administration should be considered in the analysis of the follow-up period.

The recent LOVIT trial also examined the effect of vitamin C on sepsis patients [14]. Unexpectedly, those in the vitamin C group had 1.17 times the risk of dying by 28 days than those in the placebo group. The median ICU stay was 6 days, but interestingly vitamin C was administered for only 4 days. No discussion of the biological justification for the short vitamin C administration was provided. If there is increased consumption of vitamin C in critically ill patients, it seems important to administer vitamin C over the entire period of critical illness. Furthermore, there has been concern about the possibility of harmful rebound effects if high-dose vitamin C is abruptly terminated [15–18], and such a phenomenon might be most evident in critically ill patients with very low initial vitamin C levels.

We hypothesized that the harm in the vitamin C group may be explained by the rebound effect, by abrupt termination of treatment during the period of critical illness. Furthermore, our earlier analysis showed modification of the vitamin C effect by the termination of the vitamin [13]. In this study we analyze the time distribution of deaths in the LOVIT trial. Given that the focus of our analysis is the possible short-term effects of abrupt termination of vitamin C, we restricted our analysis to the time period of the 4-day vitamin C administration and 1 week thereafter.

## Methods

The methods of the randomized double-blind LOVIT trial have been described in other papers [14, 19]. To regenerate the early part of the LOVIT survival data, we measured the steps from the LOVIT trial survival curves, figure 2 [14], by using a graphics program (see Supplementary file). Deaths over the first 11 days are shown in Table 1.

**Table 1:** Deaths in the LOVIT trial over the first 11 days.

Day	Deaths		RR (95% CI)
	Vitamin C	Placebo	
1	11	14	Days 1–4:
2	23	16	RR = 0.97 (0.65–1.5)
3	9	9	
4	6	12	
	After the end of the treatment:		
5	13	2	Days 5–7:
6	11	6	RR = 2.28 (1.24–4.2)
7	9	7	
8	5	4	Days 8–11:
9	9	4	RR = 1.51 (0.81–2.8)
10	5	4	
11	5	5	

Vitamin C was administered for 4 days, thus, from day 5 forward, the vitamin C group did not get additional vitamin C. See the Supplementary file for the explanation of data extraction from [14] for this table.

We used Cox regression to estimate the mortality rate in the vitamin C group compared with the placebo group, using the *coxph* procedure of the *survival* package of the R-project [20] to yield the rate ratio (RR) and its 95% confidence interval (CI). We used the *survSplit* procedure to split the dataset for the 2-period Cox regression analysis (Table 2). We used the likelihood ratio test to compare the fit of the single RR estimate with the two RR estimates from the 2-period Cox regression. Calculations are described in the Supplementary file.

**Table 2:** Improvement of the Cox model by allowing two separate vitamin C effects in the LOVIT trial.

Periods of vitamin C effect (days)	Improvement	
	*χ*^2^ (1 df)	*p*
0–11	Reference	
0–2 and 3–11	0.48	0.5
0–3 and 4–11	1.0	0.32
0–4 and 5–11	4.95	0.026
0–5 and 6–11	0.8	0.4
0–6 and 7–11	0.2	0.6

The improvement in the Cox regression model over the reference model (uniform effect) was tested by the likelihood ratio test. The reference model estimates that vitamin C decreased mortality uniformly over the 11 days by RR = 1.32 (95% CI 0.99–1.76; *p* = 0.060).

## Results

In the LOVIT trial, there were 429 participants in the vitamin C group and 433 in the placebo group [14]. After randomization, participants of the vitamin C group were administered vitamin C at a dose of 50 mg/kg body weight every 6 h for up to 4 days, and then vitamin C was terminated. The dosage corresponds to 16 g/day of vitamin C for an 80 kg person.

**Fig. 1:**
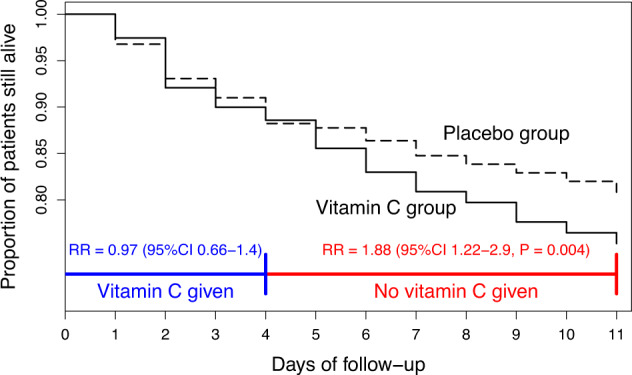
Survival curves for the LOVIT trial. The original survival curves were published with a scale up to 180 days, but such a wide scale makes it difficult to see the divergence of the vitamin C and placebo group curves after the termination of the 4-day vitamin C administration. See the Supplementary file for the calculations and Table 1 for the extracted data.

Since the focus of our analysis is the effect of abrupt termination of vitamin C, we restricted the time period of the analysis to the first 11 days, such that participants were followed for 1 week after the 4-day vitamin C administration (Table 1 and Fig. 1). Over the 11-day follow-up, there were 106 deaths in the vitamin C group, and 83 deaths in the placebo group, with RR = 1.32 (95% CI 0.99–1.76).

We tested the uniformity of the vitamin C effect by allowing separate effects of vitamin C for the early and late periods of the 11-day follow-up (Table 2). The greatest improvement in the Cox regression model appeared with the early period being 4 days (Table 2). Compared with the uniform single-size vitamin C effect over the 11-day period (RR = 1.32), the fit of this 2-period Cox regression model was significantly better (*p* = 0.026).

During the first 4 days, there were 49 deaths in the vitamin C group, and 51 deaths in the placebo group, with RR = 0.97 (95% CI 0.65–1.44). Thus, there is no evidence of harm from vitamin C *during* the period of administration.

During the week after the termination of vitamin C, there were 57 deaths in the vitamin C group, but only 32 deaths in the placebo group, with RR = 1.9 (95% CI 1.2–2.9; *p* = 0.004). Thus, there is strong evidence of harm in the vitamin C group in the week *after* the abrupt termination of vitamin C. Furthermore, there is an indication of greater harm immediately after vitamin C was terminated, compared with the later follow-up (Table 1).

Over days 5–7, there were 33 deaths in the vitamin C group, and 15 deaths in the placebo group, with RR = 2.28 (95% CI 1.24–4.2; *p* = 0.008). In comparison, during days 8–11, there were 24 deaths in the vitamin C group, and 17 deaths in the placebo group, with RR = 1.51 (95% CI 0.81–2.8). These two CIs are overlapping and do not demonstrate different effects over the two periods. However, the highly significant 2.28-fold increase in mortality during the 3 days immediately after the termination of vitamin C, with no significant evidence of harm thereafter, is consistent with the notion that it was the abrupt termination of vitamin C which caused the harm in the participants randomized to the 4-day vitamin C administration.

## Discussion

### Harm after abrupt termination of vitamin C

The LOVIT authors concluded that “in adults with sepsis receiving vasopressor therapy in the ICU, those who received intravenous vitamin C had a higher risk of death or persistent organ dysfunction at 28 days than those who received placebo” [14].

This conclusion is misleading as it implies that it was the “receiving” of intravenous vitamin C that explains the increased risk of death by day 28 in the participants randomized to vitamin C. We demonstrate that there is no evidence of difference in mortality between the trial groups *during* the 4-day intravenous vitamin C administration with RR = 0.97 (Table 1 and Fig. 1). There is, however, a significant difference between the groups immediately *after* the abrupt termination of vitamin C administration (Table 1 and Fig. 1). For a few days after the termination of vitamin C, considerable harm was observed in this group, but the two groups then approached similar death rates, consistent with the notion that the sudden termination of vitamin C caused the brief, but significant increase in mortality.

The published survival curves comparing the vitamin C and placebo groups of the LOVIT trial cover a period of 180 days [14]. Such a long follow-up makes it difficult for a reader to observe the divergence of the survival curves immediately after the 4-day vitamin C administration. Furthermore, there is no difference in mortality between the trial groups after day 30 (vitamin C: 41/267; placebo: 48/289 [14]; RR = 0.93, *p* = 0.7). Thus, it is the first 30 days which are most relevant.

Similar time-modification was previously observed in the CITRIS-ALI trial, which also examined the effect of 4-day vitamin C on sepsis. The groups differed significantly during the 4-day vitamin C administration, but not thereafter [12, 13]. Thus, in both these sepsis trials with 4-day vitamin C administration, it was the termination which significantly changed survival, although the pattern was different in the two trials.

Some medical treatments are events at a point in time, or within a narrow time period, such as vaccination and surgery. In such cases we expect long-term benefits from the short treatment and the effects can be estimated over a long follow-up period. On the other hand, many treatments require compliance over a long period of time. For example, antihypertensive and diabetes drugs are taken regularly for years before effects are seen on clinically relevant outcomes. Similarly, when vitamin C is administered for critically ill patients, it should be administered over a long period for benefits to be seen.

There is evidence that vitamin C is depleted during severe physiological stress, indicated by decreases in vitamin C levels, and parallel increases in the oxidized forms of the vitamin, i.e., dehydroascorbate and ascorbate free radical [3, 21–29]. In a healthy person, an intake of 0.1 g/day of vitamin C is enough to maintain a normal plasma level [30], but much higher doses (1–4 g/day) are needed for critically ill patients to increase vitamin C levels to within the normal range [28, 29]. The increased level of vitamin C utilization in critically ill patients does not disappear with a few doses, and may persist for the period of the critical illness.

For decades, there has been concern about the possibility of a rebound effect from abrupt termination of vitamin C [15–18]. High doses of vitamin C may increase metabolism of the vitamin. If the high dosage is terminated abruptly, the increased metabolism may lead to systemic vitamin C levels that are even lower than those prior to the high dose. This phenomenon may be particularly prominent in patients with sepsis and other critical illnesses because the consumption of vitamin C is already elevated, and systemic levels are initially low.

In the LOVIT trial, the median length of ICU stay was 6 days, that is, 2 days longer than the 4-day vitamin C administration. Furthermore, 25% of patients stayed in ICU for ≥12 days, that is ≥8 days beyond the termination of vitamin C. The LOVIT trial reported baseline vitamin C levels, but not levels thereafter. Excess mortality was pronounced in patients with the lowest baseline vitamin C levels [14], which is also consistent with the concept of harm caused by the sudden termination of high-dose vitamin C, since it is likely that the lowest levels may have decreased even further due to the rebound effect.

### Lack of benefit during vitamin C administration

The motivation for our paper was to challenge the LOVIT authors’ conclusion that it was the intravenous vitamin C that caused the higher risk of mortality or persistent organ dysfunction in the vitamin group [14]. However, refuting their conclusion does not explain the lack of observed benefit during the period of intravenous vitamin C administration. This is unexpected given the biological evidence and previous findings [1–5, 8–13]. Nevertheless, there are a few potential explanations.

In a recent retrospective cohort study conducted in Korea using propensity score matching, vitamin C administration was associated with lower mortality in sepsis patients who were treated for ≥5 days, but it was ineffective when treatment was shorter [31]. Thus, the 4-day administration of vitamin C in the LOVIT trial may have been too short for sepsis patients, one quarter of whom had ICU stays ≥12 days.

Another potentially relevant issue is the delay of vitamin C initiation. Urgency is important in the treatment of sepsis [6, 7]. If vitamin C protects against oxidative stress and other harmful consequences of sepsis, it is plausible that the effects are most pronounced when treatment is started at a very early stage. In RCTs there are likely to be delays between admission to hospital and initiation of vitamin C, due for example to the time taken for diagnosis, eligibility checks and group allocation. To our knowledge there are no data to assess the impact of delays in treatment initiation on the effects of vitamin C for sepsis. Nevertheless, urgent treatment of sepsis is possible within the RCT context [32].

Finally, mortality is not the only important outcome in the ICU context. In our view, the 862 patient LOVIT trial does not refute the significant effect of vitamin C on the length of stay in ICU found in the meta-analysis which combined 12 trials with 1766 participants [1], and the reduction in ventilation time in 8 trials with 685 patients [2]. These two meta-analyses were not restricted to sepsis studies; however, the decline in plasma vitamin C is not limited to sepsis and occurs widely in severe physiological stress [21–29].

### Ethical concerns with not treating overt vitamin C deficiency

A particular ethical concern with the LOVIT trial is that patients with severe vitamin C deficiency were not treated. In the trial protocol [19] and the trial report [14], there is no discussion of diagnosing and treating scurvy although most of the included patients had very low vitamin C levels. The Helsinki declaration states that “while the primary purpose of medical research is to generate new knowledge, this goal can never take precedence over the rights and interests of individual research subjects” [33].

Scurvy is usually associated with vitamin C levels below 0.2 mg/dl, which is 11 µM in current units. However, 11 µM should not be interpreted as a definitive level below which scurvy symptoms start to appear. For example, Hodges commented in the report of an empirical scurvy trial that “a distressing feature is the lack of precision of serum ascorbic acid levels. According to most authorities, deficiency appears after the serum level has fallen below 0.2 mg/100 ml, yet several men in these studies had obvious scurvy at a time when their serum levels were above this value” [34].

Emergence of scurvy symptoms has a much closer correlation with the total body pool of vitamin C than with plasma vitamin C level. Hodges stated that “… a comparison between plasma levels of ascorbate and pool sizes showed a very poor correlation… it is fair to say that scurvy appeared when the [vitamin C] body pool size fell below 300 mg” [35]. That said, it is not practical to measure total body pool of vitamin C in most RCTs.

A more recent study on vitamin C deprivation found that at the lowest point of depletion, with vitamin C levels of about 10 µM, mild but consistent feelings of fatigue and/or irritability were elicited [30]. These symptoms disappeared within several days of administration of 30–60 mg/day vitamin C.

Experimental vitamin C deprivation in healthy volunteers has led to dyspnea, chest pain, edema, fatigue and reduced autonomic reflexes [30, 34–38]. Case reports of scurvy have reported hypotension, tachypnea, and tachycardia [39–46], which are important symptoms of sepsis [3, 6, 7]. In the case reports, vitamin C deficiency also caused dyspnea [42–48], chest pain [42], edema [40–43, 46, 47], petecchia and ecchymoses [39–50], fatigue [39, 40, 45, 46] and musculoskeletal pains [40, 42, 47] which are also symptoms of sepsis [7]. This means that scurvy should be considered in the differential diagnosis of patients with such symptoms, in particular when plasma vitamin C levels are very low. Two of the patients in the case reports died [40, 43]. Two case reports diagnosed scurvy in patients with sepsis indicating that they can coexist [49, 50].

In the LOVIT trial, 25% of patients had baseline vitamin C levels less than 5.37 µM (figure 3 in [14]), which is half the customary level for diagnosing vitamin C deficiency based on plasma level. The upper limit in the second plasma vitamin C level quartile of LOVIT patients was 12.38 µM, which is barely higher than the usual limit for classifying vitamin C deficiency. Thus, nearly half the LOVIT patients had baseline vitamin C levels lower than the plasma level for considering scurvy, noting that scurvy symptoms often start before that plasma level has even been reached [34, 35].

Given that scurvy is a serious and potentially life-threatening disease it seems ethically inappropriate to randomize half the patients who have vitamin C levels <5.37 µM to a placebo group which is not administered vitamin C. There is no discussion in the LOVIT trial protocol or trial report [14, 19], of how the diagnosis of scurvy was intended to be carried out, or what symptoms the authors considered relevant for concluding that a patient did or did not suffer from scurvy when the vitamin C plasma level was very low.

## Conclusions

Since the publication of the results of the LOVIT trial, we are concerned about the ongoing possibility of some patients with vitamin C deficiency not being administered the vitamin on the basis that “receiving” intravenous vitamin C caused the observed harm in those randomized to the vitamin C group [14]. The LOVIT authors’ interpretation would mean that irrespective of very low vitamin levels, vitamin C should not be administered to sepsis patients. In this paper we demonstrate that it is the abrupt *stopping* of vitamin C administration that explains the observed difference between the randomized groups in the LOVIT trial, and not the *ongoing* vitamin C administration. Our interpretation is consistent with scurvy being a dangerous condition that should be avoided. Abrupt termination of high doses of vitamin C may cause a rebound effect, which can increase the harms of initially low systemic vitamin C levels.

Further research on the role of vitamin C for critically ill patients is needed and should not be discouraged by the increased mortality caused by the abrupt termination of vitamin C in the LOVIT trial. However, any future research should give thorough consideration to the ethical issues associated with randomizing patients with very low vitamin C levels (such as <11 µM) and symptoms consistent with scurvy to a placebo group, instead of treating them for vitamin C deficiency.

## Supplementary information

Abrupt termination of vitamin C from ICU patients may increase mortality: secondary analysis of the LOVIT trial

## Data Availability

Data analyzed in the paper are available in the Supplementary file and shown in Table 1.
