# Supplementary material for: Abrupt termination of vitamin C from ICU patients may increase mortality: secondary analysis of the LOVIT trial
Source: Eur J Clin Nutr. 2022 Dec 20;77(4):490–4. doi: 10.1038/s41430-022-01254-8 (PMC10115628; doi:10.1038/s41430-022-01254-8)
Supplement: Supplementary file 1 — Abrupt termination of vitamin C from ICU patients may increase mortality: secondary analysis of the LOVIT trial [file 41430_2022_1254_MOESM1_ESM.pdf]

Abrupt termination of vitamin C from ICU patients may increase mortality: secondary analysis of the LOVIT trial

Harri Hemilä and Elizabeth Chalker

Supplement
to a paper published in:
European Journal of Clinical Nutrition
https://www.nature.com/ejcn

Harri Hemilä, MD, PhD
Department of Public Health,
University of Helsinki, POB 41,
Helsinki, FI-00014, FINLAND.
E-mail: harri.hemila@helsinki.fi

2022-12-02

Contents **Page**

Figure S1: Early part of the survival curves of the LOVIT trial 2

Extraction of deaths from the survival curves over 11 days 3

Printouts of statistical calculations 4

Figure S1: Early part of the survival curves of the LOVIT trial

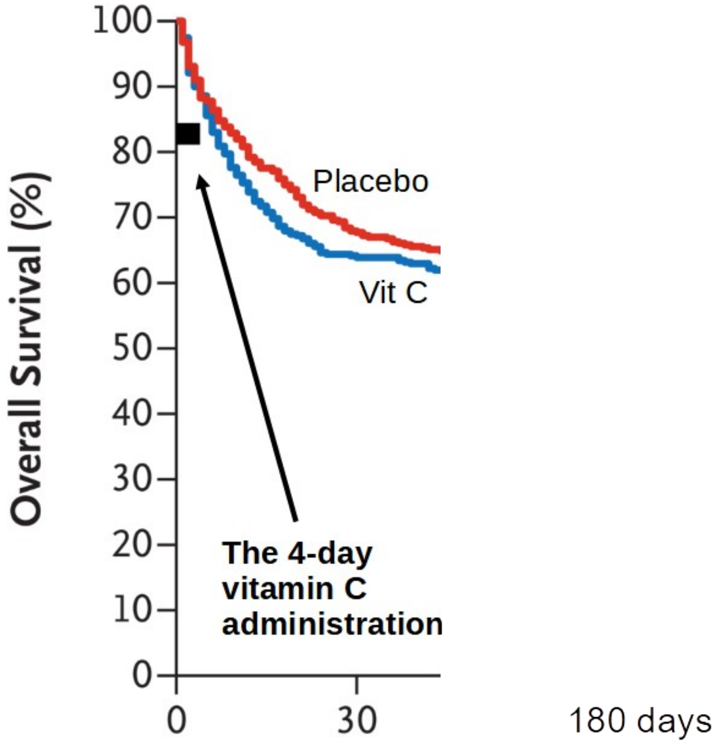

No. at Risk			
Placebo	433	289	241
Vitamin C	429	267	226

The LOVIT trial [14] published the survival curves up to day 180. In this figure, the period of 4-day vitamin C supplementation is indicated by the black box.

On day 30, there were 289 patients alive in the placebo group, and 267 in the vitamin C group.

On day 180, there were 241 patients alive in the placebo group, and 226 in the vitamin C group [14].

Thus, between 30 and 180 days there were 48 deaths in the placebo group and 41 deaths in the vitamin C group. This gives RR = 0.93 (P = 0.7; 95% CI 0.63 – 1.35).

Thus, the period from 30 days to 180 days has such few deaths that the confidence interval is very wide. Therefore the published 180-day survival curves do not provide relevant information beyond day 30. However, extension of the survival curves up to day 180 causes that the 4-day period of vitamin C administration is packed to very short segment in the survival figure. This causes that it is very difficult for a reader of the LOVIT report [14] to notice that the harm in vitamin C group occurred ***after*** the termination of the 4-day vitamin C administration, and ***not during*** vitamin C as indicated by the authors: "*those who received intravenous vitamin C had a higher risk of death or persistent organ dysfunction*" [14].

14. Lamontagne et al. N Engl J Med. 2022;386:2387-2398. https://doi.org/10.1056/nejmoa2200644

Extraction of deaths from the survival curves over 11 days

The locations of the survival curve corners were measured with a graphics program and the locations were transformed to the number of deaths per day. The extraction was restricted to the 4-day vitamin C administration and 1 week thereafter since the question was to analyze if there was change in mortality by the abrupt termination of vitamin C administration. The left-hand side of this table shows the measurements in pixels and the calculated number of patients are on the right-hand side.

0 days	30 days	pixels per day
863	1287	14.13

%	Pixels
100	128
0	1704

	Vitamin C	Placebo	Total
N:	429	433	862

Width of line	900	882	18
Width of line	Half		9

Day	Vitamin C Border	corrected	Placebo Border	corrected	Calculated Alive Vitamin C	deaths Δ/day	Alive Placebo	deaths Δ/day	Rounded Vitamin C	Placebo	Deaths per day vitC	Placebo	Day
0		128		128	429.0		433.0		429.0	433.0			0
1	160	169	171	180	417.8	11.2	418.7	14.3	418.0	419.0	11	14	1
2	262	253	229	238	395.0	22.9	402.8	15.9	395.0	403.0	23	16	2
3	295	286	261	270	386.0	9.0	394.0	8.8	386.0	394.0	9	9	3
4	300	309	305	314	379.7	6.3	381.9	12.1	380.0	382.0	6	12	4
5	365	356	313	322	366.9	12.8	379.7	2.2	367.0	380.0	13	2	5
6	405	396	334	343	356.0	10.9	373.9	5.8	356.0	374.0	11	6	6
7	438	429	360	369	347.1	9.0	366.8	7.1	347.0	367.0	9	7	7
8	457	448	375	384	341.9	5.2	362.7	4.1	342.0	363.0	5	4	8
9	490	481	389	398	332.9	9.0	358.8	3.8	333.0	359.0	9	4	9
10	509	500	404	413	327.7	5.2	354.7	4.1	328.0	355.0	5	4	10
11	527	518	422	431	322.8	4.9	349.8	4.9	323.0	350.0	5	5	11
										Days 1-4	49	51	
										Days 5-11	57	32	
										Days 1-11	106	83	

Printouts of statistical calculations

```
> table(LOVIT$day,LOVIT$dead,LOVIT$VitC)
, ,  = 0


       0   1
  1    0  14
  2    0  16
  3    0   9
  4    0  12
  5    0   2
  6    0   6
  7    0   7
  8    0   4
  9    0   4
  10   0   4
  11 350   5

, ,  = 1


       0   1
  1    0  11
  2    0  23
  3    0   9
  4    0   6
  5    0  13
  6    0  11
  7    0   9
  8    0   5
  9    0   9
  10   0   5
  11 323   5
```

```
> LOVIT_S <- Surv(LOVIT$day, LOVIT$dead)
>
> base    <- coxph(LOVIT_S ~ LOVIT$VitC, method = "efron")
```

```
> summary(base)
Call:
coxph(formula = LOVIT_S ~ LOVIT$VitC, method = "efron")

  n= 862, number of events= 189

            coef exp(coef) se(coef)     z Pr(>|z|)
LOVIT$VitC 0.276     1.318    0.147 1.88     0.06 .
---
Signif. codes:  0 '***' 0.001 '**' 0.01 '*' 0.05 '.' 0.1 ' ' 1

           exp(coef) exp(-coef) lower .95 upper .95
LOVIT$VitC      1.32      0.759     0.989      1.76

Concordance= 0.533  (se = 0.018 )
Likelihood ratio test= 3.57  on 1 df,   p=0.06
Wald test            = 3.54  on 1 df,   p=0.06
Score (logrank) test = 3.56  on 1 df,   p=0.06
```

```
> days2 <- survSplit(Surv(LOVIT$day, LOVIT$dead) ~ ., cut=c(2.5),
    episode= "tgroup", data =LOVIT)
> days2b <- coxph(Surv(tstart, tstop, event) ~ VitC:strata(tgroup),
    data=days2, method = "efron")
>
> lrtest(base,days2b)
Likelihood ratio test

Model 1: LOVIT_S ~ LOVIT$VitC
Model 2: Surv(tstart, tstop, event) ~ VitC:strata(tgroup)
  #Df LogLik Df Chisq Pr(>Chisq)
1   1  -1253
2   2  -1253  1  0.48       0.49
>
> ############
>
> days3 <- survSplit(Surv(LOVIT$day, LOVIT$dead) ~ ., cut=c(3.5),
    episode= "tgroup", data =LOVIT)
> days3b <- coxph(Surv(tstart, tstop, event) ~ VitC:strata(tgroup),
    data=days3, method = "efron")
>
> lrtest(base,days3b)
Likelihood ratio test

Model 1: LOVIT_S ~ LOVIT$VitC
Model 2: Surv(tstart, tstop, event) ~ VitC:strata(tgroup)
  #Df LogLik Df Chisq Pr(>Chisq)
1   1  -1253
2   2  -1253  1     1       0.32
>
> ############
>
> days4 <- survSplit(Surv(LOVIT$day, LOVIT$dead) ~ ., cut=c(4.5),
    episode= "tgroup", data =LOVIT)
> days4b <- coxph(Surv(tstart, tstop, event) ~ VitC:strata(tgroup),
    data=days4, method = "efron")
>
> days4b
> summary(days4b)
Call:
coxph(formula = Surv(tstart, tstop, event) ~ VitC:strata(tgroup),
    data = days4, method = "efron")

  n= 1624, number of events= 189

                              coef exp(coef) se(coef)     z Pr(>|z|)
VitC:strata(tgroup)tgroup=1 -0.0282    0.9722   0.2000 -0.14   0.8878
VitC:strata(tgroup)tgroup=2  0.6302    1.8781   0.2209  2.85   0.0043 **
---
Signif. codes:  0 '***' 0.001 '**' 0.01 '*' 0.05 '.' 0.1 ' ' 1

                            exp(coef) exp(-coef) lower .95 upper .95
VitC:strata(tgroup)tgroup=1     0.972      1.029     0.657      1.44
VitC:strata(tgroup)tgroup=2     1.878      0.532     1.218      2.90

Concordance= 0.537  (se = 0.018 )
Likelihood ratio test= 8.52  on 2 df,   p=0.01
Wald test            = 8.16  on 2 df,   p=0.02
Score (logrank) test = 8.43  on 2 df,   p=0.01
```

```
> exp(confint(days4b))
                            2.5 % 97.5 %
VitC:strata(tgroup)tgroup=1  0.66    1.4
VitC:strata(tgroup)tgroup=2  1.22    2.9
>
> lrtest(base,days4b)
Likelihood ratio test

Model 1: LOVIT_S ~ LOVIT$VitC
Model 2: Surv(tstart, tstop, event) ~ VitC:strata(tgroup)
  #Df LogLik Df Chisq Pr(>Chisq)
1   1  -1253
2   2  -1251  1  4.95      0.026 *
>
> ############
>
> days5 <- survSplit(Surv(LOVIT$day, LOVIT$dead) ~ ., cut=c(5.5),
    episode= "tgroup", data =LOVIT)
> days5b <- coxph(Surv(tstart, tstop, event) ~ VitC:strata(tgroup),
    data=days5, method = "efron")
>
> lrtest(base,days5b)
Likelihood ratio test

Model 1: LOVIT_S ~ LOVIT$VitC
Model 2: Surv(tstart, tstop, event) ~ VitC:strata(tgroup)
  #Df LogLik Df Chisq Pr(>Chisq)
1   1  -1253
2   2  -1253  1  0.82       0.36
>
> ############
>
> days6 <- survSplit(Surv(LOVIT$day, LOVIT$dead) ~ ., cut=c(6.5),
    episode= "tgroup", data =LOVIT)
> days6b <- coxph(Surv(tstart, tstop, event) ~ VitC:strata(tgroup),
    data=days6, method = "efron")
>
> lrtest(base,days6b)
Likelihood ratio test

Model 1: LOVIT_S ~ LOVIT$VitC
Model 2: Surv(tstart, tstop, event) ~ VitC:strata(tgroup)
  #Df LogLik Df Chisq Pr(>Chisq)
1   1  -1253
2   2  -1253  1  0.23       0.63
>
```

Days 5-7

```
> table(LOVIT7$day,LOVIT7$dead,LOVIT7$VitC)
, ,  = 0


       0   1
  1    0  14
  2    0  16
  3    0   9
  4    0  12
  5    0   2
  6    0   6
  7    0   7
  8    4   0
  9    4   0
  10   4   0
  11 355   0

, ,  = 1


       0   1
  1    0  11
  2    0  23
  3    0   9
  4    0   6
  5    0  13
  6    0  11
  7    0   9
  8    5   0
  9    9   0
  10   5   0
  11 328   0


> days4_7 <- survSplit(Surv(LOVIT7$day, LOVIT7$dead) ~ ., cut=c(4.5),
    episode= "tgroup", data =LOVIT7)
> days4b_7 <- coxph(Surv(tstart, tstop, event) ~ VitC:strata(tgroup),
    data=days4_7, method = "efron")
>
> summary(days4b_7)
Call:
coxph(formula = Surv(tstart, tstop, event) ~ VitC:strata(tgroup),
    data = days4_7, method = "efron")

  n= 1624, number of events= 148

                              coef exp(coef) se(coef)     z Pr(>|z|)
VitC:strata(tgroup)tgroup=1 -0.0282    0.9722   0.2000 -0.14   0.8878
VitC:strata(tgroup)tgroup=2  0.8266    2.2856   0.3114  2.65   0.0079 **
---
Signif. codes:  0 '***' 0.001 '**' 0.01 '*' 0.05 '.' 0.1 ' ' 1

                            exp(coef) exp(-coef) lower .95 upper .95
VitC:strata(tgroup)tgroup=1     0.972      1.029     0.657      1.44
VitC:strata(tgroup)tgroup=2     2.286      0.438     1.241      4.21
```

Days 8-11

```
> days7 <- survSplit(Surv(LOVIT$day, LOVIT$dead) ~ ., cut=c(7.5),
    episode= "tgroup", data =LOVIT)
> days7b <- coxph(Surv(tstart, tstop, event) ~ VitC:strata(tgroup),
    data=days7, method = "efron")
>
> summary(days7b)
Call:
coxph(formula = Surv(tstart, tstop, event) ~ VitC:strata(tgroup),
    data = days7, method = "efron")

  n= 1576, number of events= 189

                              coef exp(coef) se(coef)     z Pr(>|z|)
VitC:strata(tgroup)tgroup=1 0.237     1.268    0.165 1.44     0.15
VitC:strata(tgroup)tgroup=2 0.415     1.514    0.317 1.31     0.19

                             exp(coef) exp(-coef) lower .95 upper .95
VitC:strata(tgroup)tgroup=1      1.27      0.789     0.917      1.75
VitC:strata(tgroup)tgroup=2      1.51      0.660     0.814      2.82
```

Days 30-180

```
riskratio(41, 48, 267, 289)
           Disease Nondisease Total
Exposed         41        226   267
Nonexposed      48        241   289

    Risk ratio estimate and its significance probability

data:  41 48 267 289
p-value = 0.7
95 percent confidence interval:
 0.631 1.355
sample estimates:
[1] 0.925
```
